# Androgen deprivation therapy increases brain ageing

**DOI:** 10.18632/aging.102142

**Published:** 2019-08-02

**Authors:** Julio Plata-Bello, Ana Plata-Bello, Yaiza Pérez-Martín, Victor Fajardo, Tomás Concepción-Massip

**Affiliations:** 1 Department of Neuroscience, Hospital Universitario de Canarias, S/C de Tenerife, CP 38320, Spain; 2 Department of Urology, Hospital Universitario de Canarias, S/C de Tenerife, CP 38320, Spain

**Keywords:** prostate cancer, androgen deprivation therapy, cognitive impairment, grey matter volume, white matter lesion

## Abstract

Background: Prostate cancer (PC) is the most frequent neoplasia in the male population and androgen deprivation therapy (ADT) is frequently used in the management of the disease.

Aim: To evaluate the effect of ADT exposure on cognitive status, grey matter volume (GMV) and white matter lesion (WML) load.

Methods: Fifty ADT patients and fifteen PC-non-ADT (control) patients were included in the study. A neuropsychological evaluation was performed and a magnetic resonance imaging (MRI), with anatomical T1 and FLAIR sequences, was performed to evaluate the GMV and the WML burden.

Results: Most of the patients included in the study presented a significant cognitive impairment (CI). No significant differences were identified in the cognitive assessment between the studied groups, but when considering the educational background intragroup differences were found.

No significant difference of GMV and WML volume were identified between groups, but a negative relationship between the ADT period and the GMV was identified. Furthermore, a significant positive association between the age and the lesion volume was found in the ADT group (β=.406; p=.004).

Conclusion: PC patients exposed to ADT present an acceleration of age-related brain changes, such as WML development and GMV loss.

## Introduction

Prostate cancer (PC) is the most frequent neoplasia in the male population, affecting more than 170,000 men each year in the United States alone [1]. Most of the PC cases are currently diagnosed in localized stages, but 3-30% of cases are diagnosed in locally advanced stages or with metastatic disease [1,2]. The vast majority of PC cases are androgen-dependent [3], thus PC patients with advanced or metastatic disease are usually prescribed androgen deprivation therapy (ADT) [4]. The principle of ADT consists of the inhibition of the Gonadotrophin release hormone receptor (rGnRH) and, consequently, the decrease of the Luteinizing Hormone (LH), which is responsible for testosterone production in the

Testicles [3]. Decreasing testosterone levels has been demonstrated to increase the progression free survival and the overall survival of PC patients with advanced or metastatic disease [5].

Despite the efficacy of ADT for tumor control, this therapy is associated with the development of adverse effects which may worsen the quality of life of PC patients [6]. ADT patients are known to present a higher risk of sexual dysfunction, bone fractures, cardiovascular events, metabolic syndrome and diabetes [7].

Another adverse effect is cognitive impairment (CI), which has been extensively studied and analyzed in the last few years. Several authors described a deterioration in some cognitive functions after the onset of ADT [8,9]. The main cognitive functions that seem to be impaired during ADT are executive functions [8,9]; verbal memory [10]; and visuospatial functions [11]. ADT has also been associated with the development of dementia. In this regard, Robinson et al. (2019), in a large cohort study, described a higher risk of non-Alzheimer dementia in PC patients exposed to ADT (Hazard Ratio=1.24 (95%CI: 1.14-1.36) [12]; Nguyen et al. (2018) also described a higher risk of dementia in ADT users [7]; and Kim JH et al. (2018), in a meta-analysis of seven studies, concluded that there is a positive association between the use of ADT and the incidence of dementia (including Alzheimer’s disease) [13]. Cognitive ADT-associated effects seem to appear, at least, after 6 months of treatment [14] and seem to be positively correlated with the ADT duration [15].

However, published literature offers a certain degree of discrepancy in the putative relationship between the use of ADT and the development of cognitive impairment and/or dementia. Some authors did not find any change in cognitive assessment after one year of ADT [16]. Furthermore, Sun M et al. (2018), in a systematic review and meta-analysis, considered that the relationship between the use of ADT and the development of CI was inconclusive [17]. Nevertheless, there is enough evidence about the neuroprotective effect of androgens, thus the development of CI and/or dementia in ADT patients may be biologically plausible. In effect, androgens, especially testosterone and dihydrotestosterone, have been described as neuroprotective factors. *In vitro* data reveal a neuroprotective effect in neuron and glial cultures, with the activation of androgen receptor (AR) dependent pathways [18] and AR-independent pathways [19]. Testosterone depletion in animal models has shown to make the brain more susceptible to oxidative injury [20]. Furthermore, the neuroprotective effect of androgens has also been shown in the clinical setting. For example, it has been demonstrated that male multiple sclerosis patients with lower levels of testosterone present a more aggressive form of the disease than those with normal levels [21,22]. In the same vein, it has been shown that Alzheimer’s disease in men with low testosterone levels progresses more rapidly than in those patients with normal levels [23]. Therefore, there is a general agreement about the neuroprotective role of androgens. This neuroprotection is lost in ADT patients and this would be the explanation for the progressive cognitive deterioration.

In any case, it should be highlighted that cognitive decline is a normal condition associated with ageing. Cognitive decline normally consists of a decreasing of processing velocity, reasoning capacity and memory function. Bearing in mind that most of the PC patients are older than 65 years of age, one can consider that ADT accelerates the cognitive decline associated with ageing [11]. Age-related cognitive decline has been associated with the presence of large white matter lesions (WML) and grey matter volume (GMV) loss [24,25]. No relationship has been established between androgens and WML burden until now, but low levels of testosterone have been associated with progressive GMV atrophy, particularly in the hippocampus [26]. On the other hand, in brain diseases like multiple sclerosis, where progressive GMV loss is a common finding, the application of an androgen-based treatment has been associated with a slowing of the brain atrophy process [27]. Therefore, circulating levels of androgens may impact the GMV. This aspect has not been extensively studied in ADT patients; and neither has the possible relationship between the presence of larger WML volume and the loss of androgen neuroprotection. These two factors (i.e. GMV loss and WML burden) could be contributing to the development of the adverse ADT cognitive effects.

Therefore, the aim of the present work is to evaluate the effect of ADT exposure on the GMV and WML load of PC patients with magnetic resonance imaging (MRI), and their relationship with cognitive status.

## RESULTS

### Participants’ cognitive status

All ADT patients and 93.4% control patients (14) presented CI according to ICCTF criteria (Table 1). No significant differences in the scores of the cognitive tests were identified between the studied groups (Table 1 and Supplementary Figure 1).

**Table 1:** Clinical and neuropsychological features of the patients included in the study. Continuous variables were compared using Mann-Whitney U test, while discrete variables were compared using Chi-Square (level of significance p=.05).

	**Control** **(n=15)**	**ADT patients** **(n=50)**	**p-value**
**Age (years)**	73.4 (SD=5.9)	78.3 (SD=7.5)	.011
**Hypertension**	73.3% (11)	70.0% /35)	.540
**Diabetes**	33.3% (5)	46.0% (23)	.554
**Hypercholesterolemia**	40.0% (6)	38.0% (19)	.559
**Smoking status**			
***Active smoking***	6.7% (1)	8.0% (4)	.898
***History of smoking***	46.7% (7)	52.0% (26)	
**Metastasis**	26.7% (4)	34.0% (17)	.757
**ECOG (0-1)**	80% (12)	86% (43)	.063
**Academic degree**			
***No studies / Primary***	46.7% (7)	66.0% (33)	.148
***Secondary / Superior***	53.3% (8)	34.0% (17)	
**Education period (years)**	8.7 (SD=4.22)	8.36 (SD=4.59)	.614
**Lesion volume (cc)**	11.5 (SD=20.9)	8.3 (SD=12.6)	.684
**Grey matter volume (relative to TIV)**	.320 (SD=.13)	.365 (SD=.03)	.988
**Verbal fluency (fonetic)**			
***WLG intrusions***	1.5 (SD= 1.3)	1.6 (SD=2.1)	.657
***WLG persistence***	0.2 (SD=0.4)	0.8 (SD=1.3)	.091
**Verbal fluency (semantic)**			
***COWAT intrusions***	-	0.1 (SD=0.1)	.617
***COWAT persistence***	-	0.3 (SD=0.7)	.162
**Visuospatial and visuoperception**			
***JLOT***	18.7 (SD=7.7)	21.2 (SD=5.2)	.311
***HVOT***	16.4 (SD=7.4)	13.8 (SD=5.8)	.318
**Processing speed**			
***TMT A (time [s])***	86.7 (SD=60.8)	82.3 (SD=48.2)	.991
**Visual memory**			
***BVMT (SD)***	-0.5 (SD=1.5)	-0.7 (SD=1.7)	.646
**Verbal memory**			
***TAVEC (SD)***	-0.5 (SD=1.2)	-0.9 (SD=0.8)	.114
***TAVEC recognition (SD)***	0.2 (SD=0.9)	-0.5 (SD=1.3)	.069
**% Patients with at least 2 tests below -1.5 SD**	86.7% (13)	90.0% (45)	.514
**% Patients with at least 1 test below -2.0 SD**	93.3% (14)	100% (50)	.231
**Dependency (moderate – severe)**	26.6% (4)	22.0% (11)	.673
**Depression (moderate – severe)**	13.3% (2)	6.0% (3)	.397

The educational background was associated with a better cognitive status (intragroup differences): on the one hand, in the ADT group, the cognitive domains that show the significant differences were verbal fluency, visuospatial abilities, visual and verbal memories (Supplementary Figure 2 and Supplementary Table 1); on the other hand, in the control group, the cognitive domains that show significant differences between low- and high-educational level were visuospatial and verbal memory (Supplementary Table 1 and Supplementary Figure 1).

In the intergroup comparison, only the scores for verbal memory assessment were significantly higher in the high-level education control group than in high-level education ADT group (p=.027). The rest of the comparisons are shown in Supplementary Figure 1.

Finally, a linear regression analysis was performed to study the possible association between the score of the different cognitive tests and age, lesion burden, exposure to ADT and the possible interaction between them. A negative relationship between the score in Judgement Line Orientation Test (JLOT) and age was identified (p=0.010); and a significant interaction with ADT exposure was shown, with a significantly steeper gradient of regression in the control group as compared with the ADT patients (p=0.011) (Supplementary Table 2). Furthermore, a negative association between Hooper Organization Visual Test (HOVT) and age was identified for all patients (p=.027), but no effect was identified in the interaction with ADT exposure (Supplementary Table 2).

### Structural brain analysis: WML burden and GMV

The control group presented a mean lesion volume of 11.5 cc (SD=20.9) and the ADT group had a mean lesion volume of 8.3 cc (SD=12.6). This difference did not reach statistical significance (p=.684) (Table 1).

**Figure 1:**
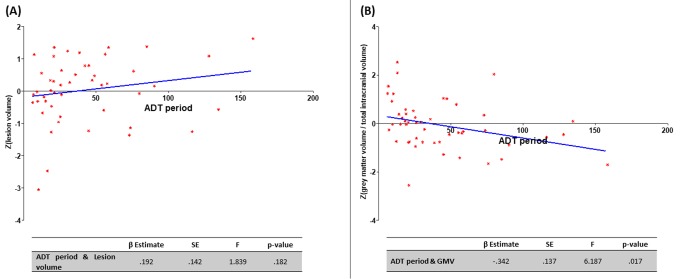
**Association of ADT period and MRI findings.** (**A**) Positive relationship between the ADT period (months) and lesion volume (no statistical significance); (**B**) negative relationship between the ADT period (months) and the grey matter volume (statistically significant; p<.05).

A univariate linear regression analysis was performed to study the possible association between ADT period, WML volume and GMV. No association between the ADT period and the WML volume was identified (β=.192; p=.182); but a negative relationship between the ADT period and the GMV was found (β=-.342; p=.017) (Figure 1).

**Figure 2:**
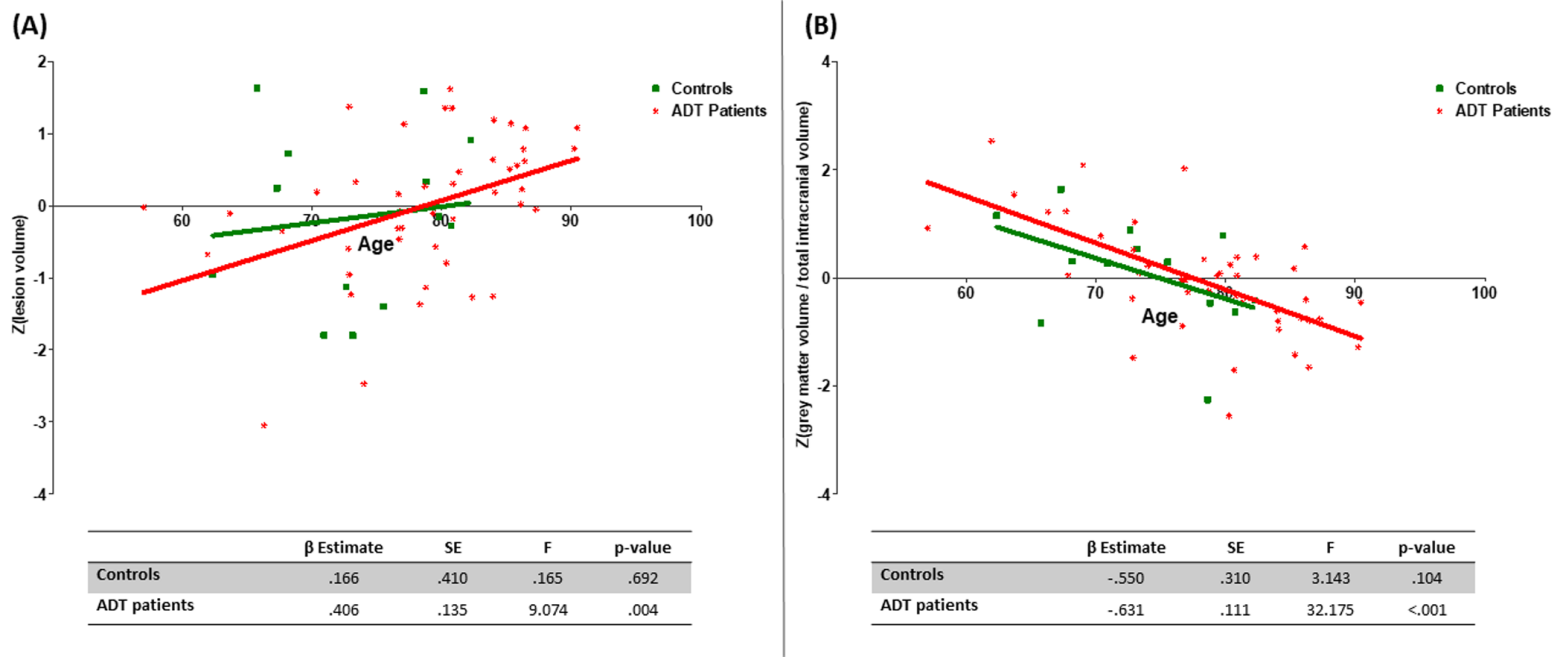
**Regression analysis between age and lesion volume** (**A**) and between the age and grey matter volume (**B**).

Subsequently, bearing in mind that age is one of the main factors associated with the appearance of WML and with GMV loss, a linear regression analysis was performed to test the effect of age on the WML volume and the GMV in controls and ADT patients. A significant positive association between age and lesion volume was found in the ADT group (β=.406; p=.004), but not in the control group (β=.166; p=.692) (Figure 2). Similarly, the ADT group showed a significant negative relationship between age and GMV (β=-.631; p<.001) but this relationship was not significant in the control group (β=-.550; p=.104) (Figure 2).

Afterwards, the main effect of the age and ADT and age-by-group interaction effect on lesion volume and GMV was calculated. Results are shown in Table 2. Age has a significant main effect on the lesion volume burden (β=.354; p=.007) and on the GM volume (β=-594; p<.001). A significant interaction of the age-by-group was also observed for both, WML volume (β=.405; p=.007) and GMV (β=-.626; p<.001). Figure 2 shows the different relationships between age and lesion volume and age and GMV in ADT patients and controls.

**Table 2:** Main and interaction effects of age and ADT on lesion volume and GMV.

	**β Estimate**	**SE**	**F**	**p-value**
**White Metter Lesion volume**				
***Age***	.354	.126	7.967	**.007**
***ADT***	.160	.326	.239	.173
***Interaction***	.405	.144	4.228	**.007**
**Grey Matter volume**				
***Age***	-.594	.102	34.131	**<.001**
***ADT***	-.169	.317	.283	.597
***Interaction***	-.626	.115	17.071	**<.001**

Since controls were younger than ADT patients, and to confirm that the differences reported above were not related with this difference, the analysis was repeated excluding those patients who were older than 80 years of age. Those over the age of 80 only belonged to the ADT group and were responsible for the difference in age distribution between groups. The effects described for WML volume were not significant, but those for GMV remained unchanged (Supplementary Figure 2).

Finally, the same analysis described above was performed by considering two different age groups (below and above the median age of the whole group: 73 years of age) (Table 3). No factor (age, group and age-by-group interaction) was found to have a significant effect when only patients <73 years old were considered. On the contrary, in patients >73 years old, age presented a significant main effect on WML volume (β=.737; p=.002) and on GMV (β=-.512; p=.014); and a significant age-by-group interaction was also found for WML volume (β=.684; p=.003) and for GMV (β=-.496; p=.020).

**Table 3:** Main and interaction effect of age and ADT on lesion volume and GMV in different age groups.

		**β Estimate**	**SE**	**F**	**p-value**
**<73 years old**	**Lesion volume**				
	*Age*	-.130	.472	.076	.787
	*ADT*	-.331	.618	.286	.601
	*Interaction*	-.058	.486	.366	.700
	**Grey Matter volume**				
	*Age*	-.663	.365	3.290	.088
	*ADT*	.267	.509	.276	.606
	*Interaction*	-.723	.375	1.980	.073
**>73 years old**	**Lesion volume**				
	*Age*	.737	.222	11.075	.002
	*ADT*	.265	.396	.447	.507
	*Interaction*	.684	.219	7.148	.003
	**Grey Matter volume**				
	*Age*	-.512	.200	6.542	.014
	*ADT*	-.066	.343	.037	.848
	*Interaction*	-.496	.204	3.358	.020

## DISCUSSION

In the present study, we have explored the differences in cognitive status and structural MRI between PC patients exposed to ADT and PC control patients. On the one hand, no significant differences were identified in the cognitive status of the studied groups, but patients with low-educational levels were more affected when receiving ADT. On the other hand, WML burden and GMV loss, which are normally associated with age, seem to be accelerated in those patients receiving ADT. A further discussion of these findings is included below.

### Cognitive status is influenced by educational level

ADT patients and controls did not statistically differ in the scores of the cognitive evaluation. In fact, controls presented a cognitive status as bad as ADT patients. In this regard, most of the patients in the present study met the ICCTF criteria for cognitive impairment. However, a differential effect of the educational level in each group was identified. The differences between low- vs. high-educational-level were more evident in the ADT group, with a larger number of cognitive domains affected. While low-educational-level patients in the control group showed worse scores than high-educational-level patients in visuospatial/visuoperceptive, processing speed and verbal memory tests; low-educational-level patients receiving ADT had lower scores than high-educational level patients in verbal fluency (phonetic), visuospatial/visuoperceptive, visual memory and verbal memory tests. Therefore, the effect of educational level, and consequently, the effect of cognitive reserve, seems to be more evident in the ADT group. Cognitive reserve explains the differences between individuals susceptible to age-related cognitive changes and pathology [29]. One of the main epidemiological factors that contributes to this reserve is educational level [30–32]. In this regard, individuals with a high cognitive reserve bear more brain damage without clinical symptoms than those with a lower cognitive reserve [33]. Low-educational level is one of the most important demographic risk factors for dementia [34]. It has been shown, for example, that cognitive reserve is able to mitigate the deleterious effect of WML in cognition [35]. Bearing this in mind, the loss of the androgen-neuroprotective effect in ADT patients seems to affect those patients with low cognitive reserve more (i.e. low-educational-level patients). In other words, the ADT patients’ brains seem to be more susceptible to the age-related cognitive decline and this decline is clinically evident in those patients with a low cognitive reserve.

This aspect may be crucial in the management of PC patients who are treated with ADT. Clinicians should consider the educational level (and other factors associated with cognitive reserve such as physical activity [36] or leisure activities [37]) when prescribing ADT. Proper recommendations about lifestyle and periodical cognitive assessment during ADT can contribute to preventing the development of a clinically significant cognitive decline and/or dementia. Furthermore, the concomitant use of some medications such as antiplatelet drugs or statins may influence the effect of ADT. In this sense, antiplatelet drug use has been associated with a protective effect against CI [38], while the use of statins has been associated with the opposite effect [39]. Therefore, the effect of the combination of these drugs with ADT on CI should be evaluated in future prospective studies.

### ADT accelerates age-related structural changes in the brain

The loss of the neuroprotective effect of androgens may also be evident when structural MRI findings are analysed. In the present work, a positive linear relationship between age and WML burden was found in ADT patients but not in controls (Figure 2). This association between androgens and WML burden has not been reported until now. Furthermore, a negative linear relationship was identified between age and GMV and the GMV was negatively correlated with the ADT period. This finding is supported by a previous work which showed that 6-months ADT patients presented a decrease in GMV in frontopolar cortex, dorsolateral prefrontal cortex and primary motor cortex, while control participants did not present such changes [40].

Both the WML and the GMV loss are common age-related findings in the elderly population. Age and hypertension are the main risk factors for the development of WML [41]. Although some authors have shown that WML burden is highly heritable [42–44], recent studies, using genome-wide association analysis, have concluded that genetic factors contribute little to WML progression in the general elderly population [45]. Moreover, Dong C et al. (2015), in the Northern Manhattan Study, associated the presence of greater WML burden with worse cognitive performance [25]. This finding has not been replicated in the present work, but the population studied here is not comparable to the one included in Dong’s study.

On the other hand, brain atrophy has been associated with cognitive performance and increased risk of stroke and dementia [24,46–48], although brain volume progressively reduced in neurologically healthy people too [49].

Androgens may have a role in these normal structural brain changes. There are consistent evidences that circulating testosterone levels decrease in an age-dependent manner and this decrease appears to be more severe in the brain [50]. Androgens regulate adult neurogenesis in the hippocampus [51] and testosterone levels have been correlated with hippocampus volume [26]. Furthermore, the effect of testosterone in GMV is also supported by clinical evidence. In this regard, Kurth et al. (2014), in an open-label phase II clinical trial, found a lack of grey matter loss (even an increase in the right frontal lobe) in multiple sclerosis men treated with testosterone compared to non-treated patients [22]. Bearing in mind the development of WML related to age, Son et al. (2016) described a higher susceptibility to oxidative injury in an animal-model brain with testosterone depletion [20]; Fanaei H et al. (2014), using a stroke rat-based model, found that the administration of testosterone was associated with a significant reduction in the infarct volume, as well as a significant increase of neurogenesis [52]; Yao et al. (2017) *in vitro* demonstrated that androgens promote the clearance of and reduce the inflammatory response induced by amyloid peptide [18]. Therefore, androgens seem to protect the brain against different injuries, and it could be hypothesized that this neuroprotective effect could also prevent the development of WML.

Bearing all the above in mind, the acceleration of testosterone decreases in PC cases that are treated with ADT may negatively contribute to the normal ageing of the brain. The increase of WML burden and GMV loss in this population may make them more susceptible to the development of cognitive impairment, mostly if they present a low cognitive reserve (as discussed above).

### Limitations

The present work has some limitations. On the one hand, transversal analysis of cognitive status and MRI findings has been done and obviously, a longitudinal study would be advisable to confirm the described cognitive and brain structural changes secondary to ADT use. This study should follow ADT patients from the beginning of the therapy and should include adequate PC and non-cancer controls. On the other hand, the selected cohorts of patients seem to be older than the mean age of PC diagnosis, thus younger patients should be included in future studies.

## CONCLUSION

PC patients receiving ADT suffer from accelerated age-related brain changes, such as WML development and GMV loss. These changes in combination with educational level (as the main factor contributing to cognitive reserve) may be associated with the development of cognitive impairment in this population.

## MATERIALS AND METHODS

### Patients

Fifty ADT patients (mean age 78.3 years [SD=7.5]) and fifteen PC-non-ADT (control) patients (mean age 73.4 years [SD=5.9]) were included in the study. All participants were right handed (using a Spanish version of the Edinburgh Handedness Inventory; http://www.neuropsicol.org/Protocol/oldfield.pdf). The patients were selected from the PC database of the Department of Urology in Hospital Universitario de Canarias (Spain). Demographic features of ADT and control groups are shown in Table 1. Age was the only variable where both groups showed a difference with statistical significance (p=.011). None of the patients included in the study had a history of exposure to other antiandrogen drugs. The criteria for selecting participants were:

### *Inclusion criteria for ADT patients*

- Diagnosis of PC with a clinical indication for ADT (leuproline, triptoreline or gosereline).

- Period exposed to ADT >= 6 months.

- Informed consent properly signed.

### *Inclusion criteria for non-ADT patients*

- Diagnosis of PC without clinical indication for ADT.

- No previous treatment with ADT.

- Informed consent properly signed.

### *Exclusion criteria*

- History of neurological or psychiatric diseases prior to de diagnosis of PC.

- History of cardiopulmonar diseases in moderate-severe stages.

- History of alcoholism or liver disease.

- History of drug abuse.

- Written informed consent was explained and signed by the patients and the control subjects. The study was approved by the Hospital Universitario de Canarias Ethics Committee, according to the Declaration of Helsinki.

### Neuropsychological assessment

A neuropsychological evaluation was performed by a specialist with 10 years of experience in neuropsychology (YPM). The evaluated functions were verbal fluency (phonetic and semantic), visuospatial and visuoperception, processing speed, visual memory and verbal memory. The tests used for the evaluation of each cognitive domain are listed in Table 4. Apart from the evaluation, the years of education and the highest educational level were registered for each patient. All assessments were performed in the morning (from 8:00AM to 12:00AM). In agreement with the recommendation of the International Cognition and Cancer Task Force (ICCTF), cognitive impairment was defined when the score of at least 2 tests was equal to or below -1.5 standard deviations (SD), or 1 test with a score equal/below -2.0 SD. A non-parametric test for two-independent sample comparison (Mann-Whitney U) was used for comparing ADT patients and Controls. Furthermore, inter- and intragroup comparisons were performed bearing in mind educational level. Finally, a univariate linear regression analysis was performed to study the possible association between a pathological score in each test and the age and/or the WML burden. The model included the presence of diabetes, hypertension, smoking status and educational level as fixed factors of no interest. Statistics were performed in SPSS v.20.0 (level of significance, p=.05).

**Table 4:** List of cognitive tests used for the cognitive assessment.

**Cognitive domain**	**Test(s)**
**Verbal fluency**	*Word List Generation (WLG)* *Controlled Oral Word Association Test (COWAT)*
**Visuospatial and visuoperceptive**	*Hooper Organization Visual Test (HOVT)* *Judgement Line Orientation Test (JLOT)*
**Processing speed**	*Trail Making Test Part A (TMT A)*
**Visual memory**	*Brief Visuospatial Memory Test (BVMT)*
**Verbal memory**	*Auditive Verbal Spanish Complutense Test (TAVEC)*
**Dependency**	*Lawton & Brody scale*
**Mood assessment**	*Beck Depression Inventory-II (BDI-II)*

### Data acquisition

MRI data was collected at the Magnetic Resonance Service for Biomedical Research of the University of La Laguna. Two imaging protocols were performed: A Fluid Attenuated Inversion Recovery (FLAIR) T2 weighted image (WI) for WML burden analysis; and finally, a T1 WI for volumetric assessment.

All images were obtained on a 3T General Electric (Milwaukee, WI, USA) scanner. The T2 FLAIR WI covered the whole brain and the acquisition parameters were: TR = 65 ms, TE = 4.50 ms, matrix size = 256 × 256 pixels, slice thickness = 2.5 mm.

The T1 WI consisted of a whole-brain three-dimensional structural image. A 3D fast spoiled gradient – recalled pulse sequence was obtained with the following acquisition parameters: TR = 10.4 ms, TE = 4.2 ms, flip angle = 20, matrix size = 512 × 512 pixels, .5 × .5 mm in plane resolution, slice thickness = 2 mm.

### FLAIR T2 WI processing and analysis

FLAIR images were processed using the Lesion Segmentation Tool for SPM (v1.2.3), using both the FLAIR and the T1 images to make the segmentation of lesions, using a threshold of K=0.3. All lesion maps were visually inspected to confirm their suitability. Using these maps, the total lesion volume of each patient was calculated. A non-parametric test for two-independent samples (Mann-Whitney U) was used to compare the lesion burden between ADT patients and controls. Furthermore, a univariate linear regression analysis was performed to study the possible association between age and the lesion burden. The model included the presence of diabetes, hypertension and smoking status as fixed factors of no interest. Statistics were performed in SPSS v.20.0 (level of significance, p=.05).

### T1 WI processing and analysis

The CAT12 toolbox (Structural Brain Mapping group, Jena University Hospital, Germany) implemented in SPM12 was used for voxel-based morphometry (VBM) analysis. All T1 WI were corrected for bias – field inhomogeneities, then spatially normalized using the DARTEL algorithm [28] and segmented into gray matter (GM), white matter (WM) and cerebrospinal fluid (CSF). The sum of the volumes of these segmented structures was considered as the total intracranial volume (TIV) and this was used to calculate the relative grey matter volume (GMV/TIV). In this manuscript, when GMV is mentioned, it refers to the relative GMV. All images were manually inspected and corrected when necessary. Apart from the non-parametric comparison between ADT patients and controls, a univariate linear regression analysis was performed to study the possible association between age and GMV. The model included the presence of diabetes, hypertension and smoking status as fixed factors of no interest. Statistics were performed in SPSS v.20.0 (level of significance, p=.05).

## Supplementary Material

Supplementary Tables

Supplementary Figures
