# Supplementary material for: Androgen deprivation therapy increases brain ageing
Source: Aging (Albany NY). 2019 Aug 2;11(15):5613–27. doi: 10.18632/aging.102142 (PMC6710035; doi:10.18632/aging.102142)
Supplement: Supplementary Tables [file aging-11-102142-s001.pdf]

SUPPLEMENTARY TABLES

Supplementary Table 1. Intragroup comparison between different educational levels. Non-parametric two sample test (Mann-Whitney U) was used (significant p<.05). The scores of cognitive tests are represented by the mean of Z-scores.

	Control group (n=15)		p-value	ADT group (n=50)		p-value
	Low-educational level	***High-educational level***		***Low-educational level***	***High-educational level***	
**Verbal fluency (phonetic)**						
WLG intrusions	0.25543	-0.44145	.126	0.23473	-0.69085	.017
WLG persistence	-0.37913	-0.3507	.931	-0.12525	0.87459	.022
**Verbal fluency (semantic)**						
COWAT intrusions	-0.13019	-0.13019	1.000	0.10985	-0.13019	.886
COWAT persistence	-0.3336	-0.3336	1.000	0.12771	0.07645	.668
**Visuospatial and visuoperception**						
JLOT	-0.67876	0.08973	.310	0.0071	0.43128	.341
HVOT	-0.37287	1.30669	.010	-0.32416	0.48956	.014
**Processing speed**						
TMT A (time [s])	0.52413	-0.57071	.048	0.11927	-0.34511	.130
**Visual memory**						
BVMT (SD)	-0.08155	0.43814	.556	-0.28614	0.50517	.029
**Verbal memory**						
TAVEC (SD)	0.41293	0.36765	.639	-0.19468	0.31948	.038
TAVEC recognition (SD)	0.14694	1.05542	.018	-0.12084	-0.01259	.825

Supplementary Table 2. Results of the linear regression analyses considering the score of each cognitive test as a dependent variable.

	β Estimate	SE	F	p-value
**WLG intrusions**				
Age	-.140	.183	.794	.450
Lesion	-021	.173	.204	.902
ADT	-.131	.436	.090	.765
*ADT * Lesion*	.318	.609	.273	.604
*Age * ADT*	.653	.420	2.424	.128
*Age * Lesion*	-.224	.178	.581	.214
*Age * ADT * Lesion*	-.060	.510	.014	.907
**WLG persistence**				
Age	.130	.184	.221	.486
Lesion	.293	.174	.446	.101
ADT	.364	.438	.690	.411
*ADT * Lesion*	.177	.613	.083	.775
*Age * ADT * Lesion*	.090	.513	.031	.861

*Age * Lesion*	.281	.179	.841	.125
*Age * ADT * Lesion*	.090	.513	.031	.861
**COWAT intrusions**				
Age	.215	.186	.236	.253
Lesion	-.348	.183	.425	.064
ADT	.269	.483	.311	.580
*ADT * Lesion*	-.348	.533	.425	.518
*Age * ADT*	.215	.443	.236	.630
*Age * Lesion*	-.328	.188	.492	.088
*Age * ADT * Lesion*	.328	.468	.492	.487
**COWAT persistence**				
Age	-.174	.192	.145	.369
Lesion	.069	.189	.016	.718
ADT	.577	.499	1.337	.254
*ADT * Lesion*	.069	.550	.016	.901
*Age * ADT*	-.174	.458	.145	.705
*Age * Lesion*	-.142	.194	.087	.467
*Age * ADT * Lesion*	.142	.483	.087	.770
**JLOT**				
Age	-.047	.159	8.299	.768
Lesion	.153	.156	3.252	.331
ADT	1.211	.447	7.342	**.010***
*ADT * Lesion*	-.506	.456	1.281	.264
*Age * ADT*	1.099	.414	7.039	**.011***
*Age * Lesion*	.002	.160	1.835	.988
*Age * ADT * Lesion*	-.558	.415	1.804	.187
**HVOT**				
Age	-.337	.147	11.937	**.027***
Lesion	.023	.144	.375	.872
ADT	.276	.358	.594	.445
*ADT * Lesion*	.287	.393	.534	.469
*Age * ADT*	.529	.348	2.309	.136
*Age * Lesion*	.068	.149	.027	.648
*Age * ADT * Lesion*	.077	.363	.045	.832
**TMT A**				
Age	.173	.165	5.356	.298
Lesion	.125	.156	.764	.428
ADT	-.539	.386	1.949	.171
*ADT * Lesion*	-.120	.422	.080	.778
*Age * ADT*	-.525	.377	1.941	.171
*Age * Lesion*	.043	.160	.129	.791
*Age * ADT * Lesion*	-.055	.390	.020	.888
**BVMT**				
Age	-.013	.197	1.809	.947
Lesion	.100	.203	.058	.625
ADT	.035	.573	.004	.952
*ADT * Lesion*	.329	.534	.381	.542
*Age * ADT*	.650	.503	1.671	.206
*Age * Lesion*	.014	.187	.809	.942
*Age * ADT * Lesion*	.454	.474	.916	.346
**TAVEC (SD)**				
Age	.123	.154	.054	.427
Lesion	-.135	.151	.141	.377
ADT	-.435	.379	1.322	.256
*ADT * Lesion*	-.426	.416	1.049	.311
*Age * ADT*	.161	.368	.192	.663
*Age * Lesion*	.004	.154	.042	.980
*Age * ADT * Lesion*	-.071	.383	.035	.853
**TAVEC**				
Age	-.010	.170	.898	.956
Lesion	-.090	.168	.044	.594
ADT	-.458	.417	1.207	.278
*ADT * Lesion*	-.084	.458	.033	.856
*Age * ADT*	.365	.406	.811	.373
*Age * Lesion*	.235	.172	.355	.179
*Age * ADT * Lesion*	.218	.423	.266	.609
