# Supplementary material for: Androgen deprivation therapy increases brain ageing
Source: Aging (Albany NY). 2019 Aug 2;11(15):5613–27. doi: 10.18632/aging.102142 (PMC6710035; doi:10.18632/aging.102142)
Supplement: Supplementary Figures [file aging-11-102142-s002.pdf]

SUPPLEMENTARY FIGURES

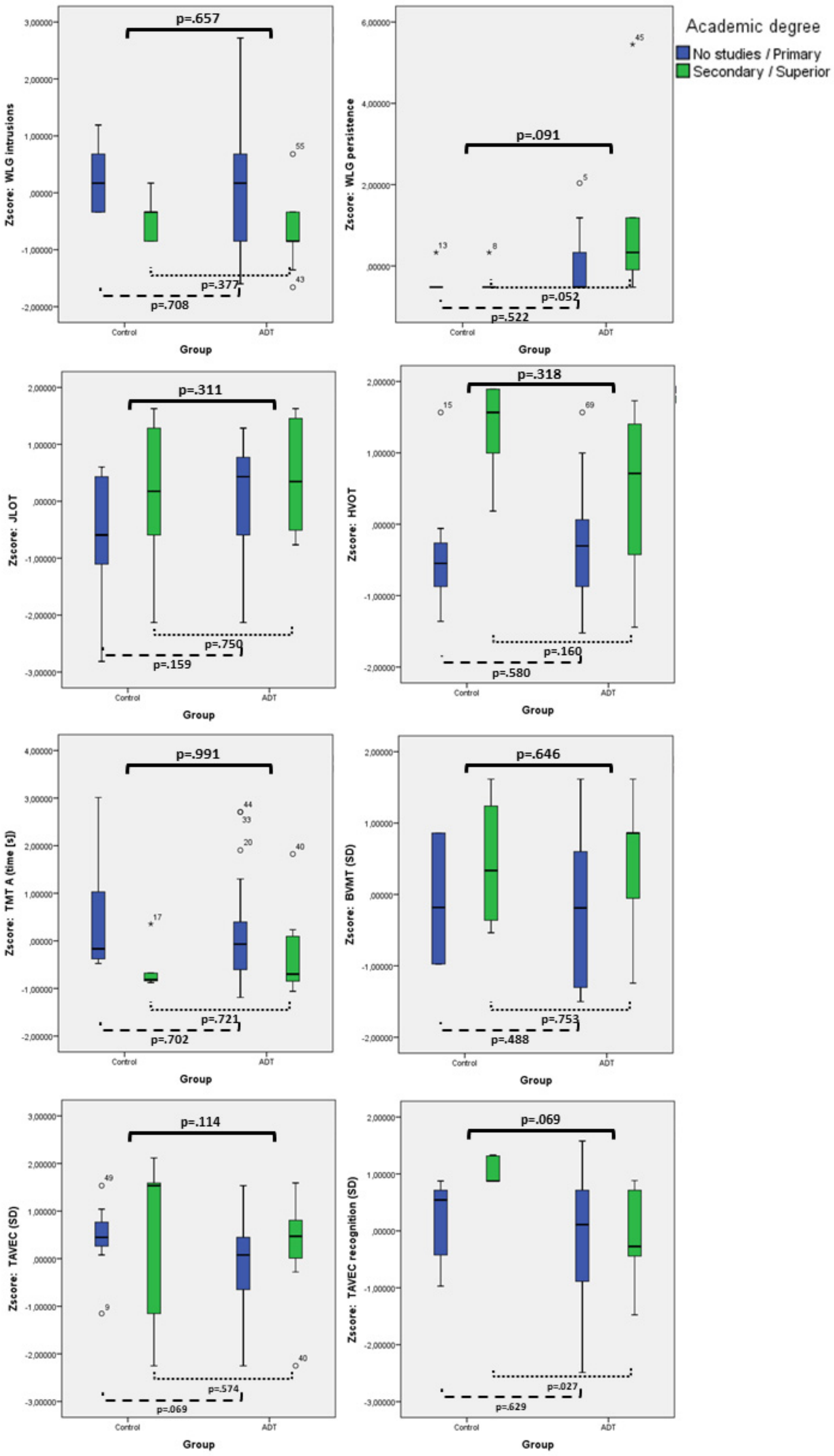

Supplementary Figure 1. Intergroup comparison between control and ADT groups. Upper p-value represents the level of significance of the comparison between control and ADT group without considering educational level. Lower p-values represent the level of significance between control and ADT groups with low-educational level (discontinuous line) and with high-educational level (dotted line).

(A)

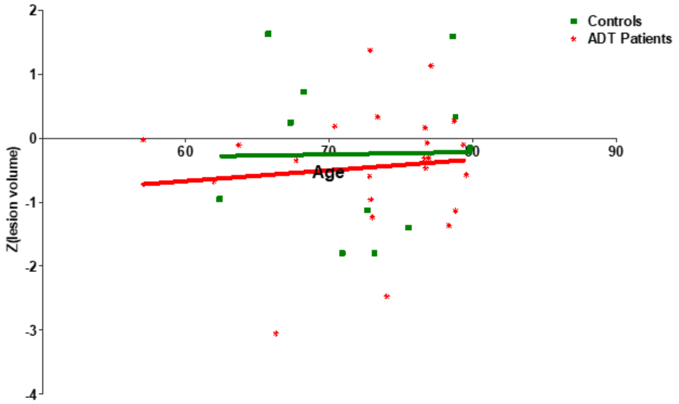

	β Estimate	SE	F	p-value
Age	.255	.203	1.574	.217
ADT	.017	.379	.002	.964
Interaction	.336	.238	.998	.166

(B)

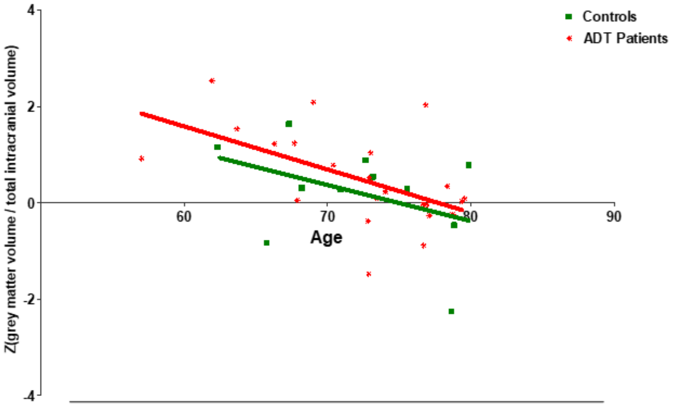

	β Estimate	SE	F	p-value
Age	-.686	.165	17.321	<.001
ADT	-.089	.363	.061	.807
Interaction	-.758	.189	.8.999	<.001

Supplementary Figure 2. Main effect of age and ADT and age-by-group interaction effect on lesion volume and GMV considering only patients younger than 80 years of age.
